# Identifying and Characterizing Types of Balance Recovery Strategies Among Females and Males to Prevent Injuries in Free-Standing Public Transport Passengers

**DOI:** 10.3389/fbioe.2021.670498

**Published:** 2021-07-05

**Authors:** Jia-Cheng Xu, Ary P. Silvano, Arne Keller, Simon Krašna, Robert Thomson, Corina Klug, Astrid Linder

**Affiliations:** ^1^Swedish National Road and Transport Research Institute, Linköping, Sweden; ^2^AGU Zürich, Zurich, Switzerland; ^3^Faculty of Mechanical Engineering, University of Ljubljana, Ljubljana, Slovenia; ^4^Mechanics and Maritime Science, Chalmers University of Technology, Gothenburg, Sweden; ^5^Vehicle Safety Institute, Graz University of Technology, Graz, Austria

**Keywords:** balance strategy, balance recovery, free-standing passengers, human balance, perturbation, public transport, step strategy

## Abstract

Free-standing passengers on public transport are subjected to perturbations during non-collision incidents caused by driver maneuvers, increasing the risk of injury. In the literature, the step strategy is described as a recovery strategy during severe perturbations. However, stepping strategies increase body displacement, ultimately subjecting passengers to higher risk of impacts and falls on public transport. This study investigates the influence of different recovery strategies on the outcome of balance recovery of free-standing public transport passengers, challenged in postural balance by the non-uniform vehicle dynamics. From high-speed video recordings, a qualitative investigation of the balance responses of volunteer participants in a laboratory experiment was provided. On a linearly moving platform, 24 healthy volunteers (11 females and 13 males) were subjected to perturbation profiles of different magnitude, shape and direction, mimicking the typical acceleration and deceleration behavior of a bus. A methodology categorizing the balancing reaction to an initial strategy and a recovery strategy, was used to qualitatively identify, characterize and, evaluate the different balance strategies. The effectiveness of different strategies was assessed with a grading criterion. Statistical analysis based on these ordinal data was provided. The results show that the current definition in the literature of the step strategy is too primitive to describe the different identified recovery strategies. In the volunteers with the most successful balancing outcome, a particularly effective balance recovery strategy not yet described in the literature was identified, labeled the *fighting stance*. High jerk perturbations seemed to induce faster and more successful balance recovery, mainly for those adopting the fighting stance, compared to the high acceleration and braking perturbation profiles. Compared to the pure step strategy, the characteristics of the *fighting stance* seem to increase the ability to withstand higher perturbations by increasing postural stability to limit body displacement.

## Introduction

Public transport is considered a safe mode of transportation. However, standing passengers on buses and trams are subjected to perturbations due to vehicle maneuvers that might cause injuries. The risk of injury due to falling in non-collision incidents on public transport has been estimated in a meta-analysis to be between 0.2 and 0.3 per million passenger km (Elvik, 2019). Factors contributing to the risk of falling include the perturbation profile (magnitude, duration, and orientation) and passenger capabilities (balance recovery, age, gender, and health condition). The literature highlights that harsh acceleration and sudden braking perturbations are important contributing factors, and that the group of female passengers aged 65+ are overrepresented in non-collision incidents on public transport (Kirk et al., 2003; Albertsson and Falkmer, 2005; Björnstig et al., 2005; Halpern et al., 2005). Furthermore, in a more recent study, Silvano and Ohlin (2019) found that female involvement is also high for other age groups with 87 and 86% involvement for the age group brackets of 16–24 and 25–65, respectively.

Postural balance is often described in terms of three fundamental balance strategies: (1) the ankle, (2) the hip, and (3) the step strategy (Nashner and McCollum, 1985; Winter, 2009). Another strategy found in the literature, yet not so extensively used, is the squat strategy, which incorporates both knee and hip flexion for stability (Hemami et al., 2006; Cheng, 2016). The ankle and hip strategies are fixed-support strategies, while the step strategy is a change-in-support (CIS) strategy induced during more severe perturbations as the center-of-mass (CoM) and base-of-support (BoS) are displaced due to the momentum of the perturbation. The BoS is defined as the area under and between the feet. To maintain the full-body system in balance, the CoM projecting on the floor must be within the BoS to maintain equilibrium. For less severe perturbations, the combination of ankle and hip adjustments is usually sufficient to maintain balance. Change-in-support strategies with single or multiple recovery steps are the most dominant strategies to avoid falls, by shifting the BoS to contain the displaced CoM (Maki and McIlroy, 1997; Maki et al., 2008). Multiple-step strategies have been shown to result in less effective balance recovery, compared to single-step recovery in translational perturbations (Robert et al., 2007; Carty et al., 2011; Barrett et al., 2012; Carty et al., 2012a, b; Mille et al., 2013; Crenshaw and Kaufman, 2014; Carty et al., 2015), and lateral perturbations (Mille et al., 2005, 2013; Hilliard et al., 2008; Bair et al., 2016; de Kam et al., 2017; Borrelli et al., 2021). Single-step responses are characterized by longer step lengths and shorter initiation times, usually utilized by younger subjects, and Cronin et al. (2013) suggested that a single-step strategy can be assumed as the most optimal response. This is biomechanically efficient, as a larger step increases balance recovery by relocating the stepping foot ahead of the CoM and generates larger contact forces between the foot and the ground (King et al., 2005). In contrast, older adults tend to execute a multiple-step strategy (Luchies et al., 1994; McIlroy and Maki, 1996; Hsiao and Robinovitch, 1999). However, increased step length and shorter step initiation time is observed for both younger and older subjects (Do et al., 1982; Luchies et al., 1994; Maki et al., 1996; Thelen et al., 1997; Hsiao and Robinovitch, 1999; Wojcik et al., 1999). This has been experimentally confirmed by measuring release angles to recover a stable upright stance with a single step, where recovery increased through larger and quicker steps, among both young and elderly women (Hsiao-Wecksler and Robinovitch, 2007). However, during more severe perturbations, multiple-step responses are natural and can be executed in various ways (Maki and McIlroy, 1996; Hsiao and Robinovitch, 1999). As multiple stepping increases body displacement and the risk of impacts with interior design or passengers on public transport, it can be hypothesized that recovery strategies increase dynamic postural stability with different effectiveness.

Tether-release methods to simulate trips and slips, for fall prediction, are very common in the literature (Thelen et al., 1997; Hsiao and Robinovitch, 1999, 2001; Cyr and Smeesters, 2007; Carty et al., 2011; Cheng, 2016; Okubo et al., 2019). This kind of experimental setup provides lean angle thresholds to study the difference between single- and multiple-step strategies to avoid falls, where single-step responses are used to identify perturbation threshold limits to successfully recover balance (Hsiao-Wecksler and Robinovitch, 2007; Barrett et al., 2012; Carbonneau and Smeesters, 2014; Carty et al., 2015). Graham et al. (2014) instructed younger and older volunteers to recover balance using a single step to model the muscle contribution for recovery, and the recovery strategy of older multiple steppers was considered less effective than for older single steppers. Hence, single-step strategies seem to be advantageous over multiple-step strategies, arguably important on public transport to avoid, e.g., head impacts due to increased body displacement (Robert et al., 2007). Pull perturbations (waist or shoulder) in multiple directions are also common to study stepping responses in a similar manner (Pai et al., 1998; Sturnieks et al., 2013; Fujimoto et al., 2015, 2017; Bair et al., 2016; Verniba and Gage, 2020).

Studies conducted with translational perturbations on a moving platform, which would be the most realistic laboratory setup to simulate a standing passenger on public transport, are less common due to the more complicated setups. These perturbation studies usually evaluate the stepping response limited to identification, i.e., only differentiating between individual responses, of single- and multiple stepping with minor specific illustration or description of the different executions (Rogers et al., 1996; Mille et al., 2005; Robert et al., 2007; Carty et al., 2012b; Lee et al., 2014; Honeycutt et al., 2016; de Kam et al., 2018; Borrelli et al., 2019). Furthermore, stepping responses comparing older to younger adults are also common since older adults constitute the most vulnerable age group to lose balance during platform perturbations (Brauer et al., 2002). Instead, the aforementioned studies, regardless of the perturbation type, characterize the stepping responses based on quantitative measures such as step initiation times, number of recovery steps, CoM or CoP kinematics, and margin of stability (Hof et al., 2005; Hof and Curtze, 2016). Ideally, qualitative identification and characterization of different stepping responses could complement such quantitative measures, since multiple step responses can have different effectiveness and execution. More importantly, since instructing volunteers to recover balance using a single step is considered as the most effective strategy, characterizations of the single-step execution might also be vastly different among different age groups and genders. To the authors’ best knowledge, there have been a few studies that have identified and characterized strategies in more detail than single- and multiple stepping (Eng et al., 1994; Cordero et al., 2003; de Boer et al., 2010; Mille et al., 2013; Krasovsky et al., 2014; Honeycutt et al., 2016; Karekla and Tyler, 2018a). For example, de Boer et al. (2010) characterized and confirmed previous findings (Eng et al., 1994; Cordero et al., 2003), regarding an elevating and a lowering strategy during induced stumble perturbations. Honeycutt et al. (2016) sought to identify main kinematic characteristics of stroke survivors’ stepping responses, characterizing two additional compensatory step strategies (called “pivot” and “hopping”) utilized to avoid falls beside the traditional pure step strategy.

Further characterization of the step strategy exists, but the perturbation levels used in the literature are rarely similar to those experienced on public transport. Robert et al. (2007) conducted linear sled perturbations to simulate emergency braking and a low collision scenario to study head excursion in three different starting positions (free-standing, backrest, and holding a vertical bar) and found a main and an alternative strategy. However, these strategies were differentiated mainly by head kinematics and not stepping characteristics. Schubert et al. (2017) subjected older passengers in standing upright postures to acceleration profiles similar to those encountered during regular start and stop maneuvers in traffic, measuring ground reaction and handgrip forces. Although grasping strategies, i.e., using hand support such as handrails to recover balance, is effective, it does not account for free-standing scenarios when handrails are out of reach. Karekla and Tyler (2018a; 2018b) analyzed stepping responses during normal gait without handrails of moving passengers inside an accelerating bus to determine perturbation thresholds with respect to standing postural balance. Here, some characterization of step responses between males and females were found based on number of steps. In that study, a recommended threshold of 2.0 m/s^2^ to account for balance of all passengers using handrails, 1.0 m/s^2^ to account for free-standing postures, and 1.5 m/s^2^ for the majority of younger passengers during normal gait (Karekla, 2016; Karekla and Tyler, 2018a, b). These levels are commonly exceeded in regular operation of public transport (Karekla, 2016; Karekla and Tyler, 2018a, b). However, this was not in free-standing scenarios and only acceleration levels were considered with no jerk variations.

The literature assessing stepping responses during perturbations is very extensive, but a gap was identified between the literature on recovery strategies and different perturbation characteristics causing balance instability on public transport. Identifying balance strategies when subjected to perturbation profiles similar to those on public transport complements current literature on stepping strategies. Characterizing such stepping responses might provide insight on how effective balance recovery in free-standing scenarios can be executed, to benefit passengers. It might also provide insight on how to optimize vehicle dynamics for passenger safety and discomfort, especially with the development of automated vehicles for public transport. Therefore, the aim of this study was to provide a first investigation to identify and characterize recovery stepping strategies that healthy free-standing females and males display during perturbations of different characteristics, mimicking relatively strong bus accelerations and decelerations. The identified strategies were analyzed for their effectiveness in balance recovery by a qualitative measurement, to mainly provide insight on different CIS strategies and understand perturbation thresholds.

## Materials and Methods

**FIGURE 1:**
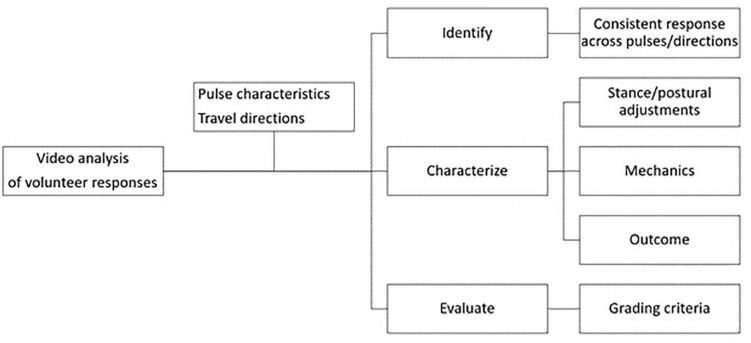
Analysis framework.

The methodology used for the analysis of the balance strategies was based on visual analysis of video recordings of dynamic tests with volunteers, where the standing participants were exposed to translational acceleration/deceleration perturbations. It comprises three steps: (i) identification, aiming at distinguishing different strategies used among the volunteers; (ii) characterization, describing the execution and characteristics of the identified strategies biomechanically, and (iii) evaluation, with the aim of systematically assessing the effectiveness of the identified and characterized strategies. The methodological framework is, therefore, based on a visual grading experiment approach which has been used in other fields such as clinical experiments and radiography (Ivanauskaite et al., 2008; Smedby and Fredrikson, 2010). The methodology is depicted in Figure 1 below.

### Volunteer Tests

**FIGURE 2:**
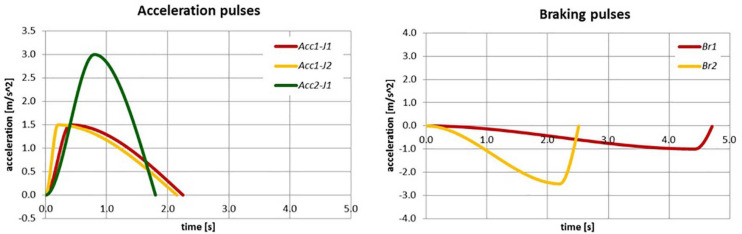
Pulse profile characteristics of acceleration and braking pulses.

The experiment was conducted on 24 healthy volunteers (11 females and 13 males) close to the 50th percentile stature, see Tables 1, 2. While standing on a moving platform, the participants were exposed to five different acceleration profiles designed to mimic the behavior of buses in normal operation (see Figure 2 and Table 3). The acceleration pulses is described in more detail in Linder et al. (2020). Each perturbation was tested both in forward and rearward direction, i.e., the participant either facing the direction of travel or the opposite direction. The volunteers were instructed to initially adopt a relaxed standing posture, feet hip-wide apart, on a designated spot on the platform, while trying to withstand the perturbation without grabbing any parts of the platform. For the safety of the participants, the platform was partly padded, and they were attached to a harness system to prevent them from falling off the platform. The tests were monitored laterally and transversally by two high speed cameras (VEO 640L, Vision Research, Wayne, NJ, USA), the footage of which the analysis in this article is based on.

**TABLE 1:** Volunteer information.

No.	Gender	Age	Height (cm)	Weight (kg)	No.	Gender	Age	Height (cm)	Weight (kg)
1	M	37	185.0	83.3	13	F	23	178.5	68.7
2	M	42	191.5	110.5	14	F	25	167.0	79.8
3	M	63	177.0	102.7	15	F	33	160.0	80.3
4	F	30	168.0	57.5	16	F	38	170.5	54.6
5	F	38	161.0	54.7	17	M	24	174.0	75.5
6	F	34	165.0	58.6	18	M	32	173.0	78.6
7	M	40	179.0	84.2	19	M	30	171.0	82.9
8	F	22	155.0	53.6	20	M	34	182.0	83.4
9	F	28	168.0	64.0	21	M	44	180.0	103.8
10	F	46	167.5	67.5	22	M	35	180.0	75.7
11	M	21	180.0	79.5	23	M	30	181.5	86.0
12	M	30	176.0	74.1	24	F	31	160.0	72.6

**TABLE 2:** Age, height, and weight summary statistics and gender.

Description	Mean ± SD	Minimum	Maximum
Age	33.8 ± 9.0	21	63
Female Age	31.6 ± 7.2	22	46
Male Age	35.5 ± 10.6	21	63
Height (cm)	172.9 ± 9.2	155	191.5
Female height (cm)	166.5 ± 6.4	155	178.5
Male height (cm)	179.2 ± 5.4	171	191.5
Weight (kg)	76.3 ± 15.3	53.6	110.5
Female weight (kg)	64.7 ± 9.9	53.6	80.3
Male weight (kg)	86.2 ± 11.8	74.1	110.5

**TABLE 3:** Main characteristics of the perturbation profiles used.

Consecutive trial	Profile name	Magnitude (m/s^2^)	Rise time (s)	Duration (s)	Jerk (m/s^3^)
1	Lowest braking (Br1)	1.0	4.43	4.72	0.3
2	Baseline (Acc1-J1)	1.5	0.4	2.25	5.6
3	Highest jerk (Acc1-J2)	1.5	0.2	2.15	11.3
4	Highest acceleration (Acc2-J1)	3.0	0.8	1.8	5.6
5	Highest braking (Br2)	2.5	2.2	2.9	1.7

### Identification of Different Balance Strategies

To identify the different balance recovery strategies among the volunteers, the balance response of each volunteer during a specific perturbation trial was categorized into two phases:

#### Initial Phase

Indicated by the first balancing reactions occurring in the starting position when subjected to a perturbation. Hence, this is the first balance strategy executed by the volunteer. It is described by the fixed-support strategies, the ankle, hip, and squat strategies, since the BoS is stationary at this point and the CoM is displaced from its equilibrium at the start of the perturbation (causing an initial balance instability). If the perturbation is not severe, balance can be maintained by a fixed support strategy.

#### Recovery Phase

Defined as the phase where a recovery strategy, induced as the severity of the perturbation increases for the volunteer. Here, the initial strategy was not sufficient to maintain a stationary BoS. The balance instability displaces the CoM and BoS beyond static equilibrium limits and a CIS strategy (step strategy) was utilized to attempt to counteract the perturbation. The step strategy is utilized to keep the CoM within the translating BoS.

The hypothesis suggests that the step strategy is too primitive to characterize the differences in the balance responses among the volunteers. Therefore, different recovery strategies were defined, because they determine whether a volunteer is successful in withstanding the perturbation or not. Furthermore, the characterization of a specific recovery strategy was evaluated in relation to its balancing effectiveness, i.e., the resulting balance outcome during a specific perturbation, as a result of utilizing a successful recovery strategy. The characterization methodology describes how the step strategy adopted during balance instability enabled differentiation of the identified balance strategies.

### Characterization of Balance Recovery Strategies

Exceeding the initial balance maintenance strategy activates a recovery strategy and initiates the CIS strategy in humans. Two reactions can occur without falling: (i) balance recovery occurs through returning to a fixed-support strategy (stationary BoS), or (ii) a continuous CIS strategy is applied (the BoS is translating beyond static equilibrium limits). A stable position (stationary BoS) represents body control (withstanding the perturbation) and increases stability (balance equilibrium), maintaining or recovering balance. When a step strategy is adopted to counteract the instability produced by perturbation, compensatory stepping is utilized to keep the CoM within the BoS, as the latter is displaced when the body is perturbed. A recovery strategy was established based on the identification of different balance strategies during each perturbation trial, using the factors denoted below in bold.

To regain a stationary BoS and, consequently, body control and the stability to counteract the momentum from the perturbation, a new posture is required. The identified new posture has been defined as a **stance**, i.e., a fixed-support strategy utilized to minimize continued compensatory stepping. Harness deployment resulting in a stance is not considered a successful recovery strategy. On the other hand, continuous **postural adjustments** denote movements to maintain or recover balance, i.e., taking compensatory steps to recover balance equilibrium when a stationary BoS cannot be achieved or maintained during a perturbed state.

The **mechanics** of the balance recovery can be described in terms of the BoS and the CoM during the perturbation. An effective strategy should allow the CoM to be within the BoS throughout the movement, to limit BoS translation from the starting position due to controlled CoM displacement. Minimal total translation from the starting position was considered ideal to display active counteraction to withstand the perturbation, resulting in balance recovery from a perturbed balancing state. A stable position, i.e., an efficient stance, facilitates balance equilibrium of the CoM and BoS, indicating that these components are not translating, and the volunteer has achieved balance equilibrium or returned to the starting position (through stepping) and has counteracted the perturbation. Postural adjustments denote continuous balance instability, where each adjustment is counteracting a perturbed balance equilibrium.

Strategy **outcome** identifies the effectiveness of the response in terms of balance recovery from perturbed states. If a combination of stance and/or postural adjustments allows the “mechanics” to act and turn a perturbed state into a state of balance equilibrium, then the strategy outcome is considered successful. Given that a twofold classification “successful or not” in some cases would be too primitive to describe the strategy outcome, the following three categories were applied: effective, less effective, and ineffective.

### Evaluation of Balance Recovery

To enable differentiation of different stepping strategies and evaluate the balance recovery qualitatively, a simple ranking system was developed to enable comparison of responses among the different perturbations. A statistical analysis based on the qualitative evaluation (ordinal data) is provided to understand the difference between genders and identified strategies.

To understand and interpret the identified strategies, a grading system was established to analyze the outcome of the different strategies utilized during a perturbation to recover/maintain balance. This is an ordinal scale of effectiveness, i.e., balance responses were scaled to obtain so-called ordinal data (Merbitz et al., 1989). Different criteria constitute the grading system used during the video analysis when the volunteers were subjected to perturbation. A grading scale of 2 (*effective*), 1 (*less effective*), or 0 (*ineffective*) points were used to determine the effectiveness of the adopted strategy when analyzing a pulse trial. The properties defined in Section “Characterization of Balance Recovery Strategies” were used for the evaluation.

If the volunteer managed to hold a stable position (stance) or return to the starting position (fully controlled step strategy), then the strategy has been considered effective. The postural adjustments were deemed “*effective”* if it was evident that the volunteer had recovered balance, and displayed control through compensatory movements, to counteract additional perturbed balance to recover balance equilibrium. In contrast, the strategy was deemed *“ineffective”* if the volunteer showed instability in the stance or postural adjustments, and exhibited an unstable balancing state, i.e., continuous compensatory stepping representing difficulties in counteracting the perturbation, or harness deployment). However, a strategy would be *“less effective”* if balance has been achieved yet showing some instability or constant utilization of compensatory steps or adjustments throughout the perturbation. Displayed instability through compensatory stepping has been considered to increase the risk of harness deployment and is therefore not ideal inside a public transport vehicle to avoid the risk of impacts. Table 4 below, defines the grading which represents the effectiveness of each balance strategy based on the outcome during a perturbation.

**TABLE 4:** Grading table for balance recovery based on the characterization of strategy effectiveness.

Assessment	Effective (2 points)	Less effective (1 point)	Ineffective (0 point)
Stance	Finds and keeps a firm stance Stable position	Difficulties finding and keeping a stance Less stable position	Unable finding and keeping a stance Unstable position
Postural adjustment	Minimal body adjustments Body control	Body adjustments Less body control	Major body adjustments No body control
Mechanics	CoM within the BoS Minimal translation of CoM and BoS	CoM slightly outside BoS (action: compensatory stepping to try to maintain CoM within BoS) Some translation of CoM and BoS	CoM outside the BoS (rigorous stepping, difficulties in maintaining stable CoM within BoS, exhibiting many difficulties during a trial) Larger translation of CoM and BoS
Outcome	Firm and stable stance or returning to the starting position Few compensatory steps Clear body control and stability	Less firm and stable stance and/or multiple compensatory steps Displayed instability, some body control	Harness deployment No clear body control or stability

#### Gender Comparisons

It is well known that there are anthropometric differences between females and males, generally more evident in terms of height, musculature and fat mass, to name a few (Schneider et al., 1983; Al-Haboubi, 1998; Glenmark et al., 2004; Schorr et al., 2018). Physical capabilities, either through gender and anthropometrical differences or athletic background and experience, might affect the execution of a balance strategy. Thus, from the identification and evaluation of balance strategies, gender differences have been examined to understand the effectiveness of utilized strategies and their execution.

#### Pulse Severity

The volunteer tests provided the opportunity to analyze how the different perturbation characteristics (see Figure 2 and Table 3) disturb a standing passengers’ equilibrium and how passengers counteract the disturbance. This was achieved by analyzing and comparing the balance recovery and reaction strategies among the volunteers for the different perturbation profiles. In order to reduce the risk of injury to standing passengers, the success and failure ratios of the volunteers due to the different pulse severities, have been estimated to identify the most challenging perturbations to understand the magnitude thresholds.

#### Statistical Analysis

In addition to the qualitative evaluation, statistical tests were carried out on the ranked score data (so-called ordinal data) to evaluate whether there are statistical differences among the identified strategies or between genders. The non-parametric (Kruskal and Wallis, 1952) was applied for ordinal data at 5% level of significance. The Null hypothesis is that the sample distributions come from the same population, Whereas the alternative hypothesis states that the distributions are from different populations.

## Results

The following subsections describe the tabulated results that constitute the findings of the identification of different balance strategies, characterization of these strategies, and how the recovery strategy of the volunteers affected balancing outcome.

### Identification and Characterization of Balance Strategies

From the qualitative video analysis, different strategies were identified. Different execution of similar strategies was found among the volunteers, also between the genders. Supplementary Appendix 1 shows the identified strategies for each volunteer during each perturbation trial, categorized into the characterization of an initial and a recovery strategy. The overall initial and recovery strategies were determined based on the most frequently used strategy by a volunteer. In Table 5, the volunteers were ranked based on their performance according to the grading criteria. The different perturbations affected the volunteers’ responses, and the different pulse severities have been highlighted in the columns of Supplementary Appendix 1. The success and failure rate presents how well the volunteers performed as a group and also denotes the most challenging perturbation.

**TABLE 5:** Tabulated grading of balance recovery during each perturbation and ranking of the volunteer outcome based on strategy effectiveness (M, male; F, female).

Volunteers	Gender	Initial strategy	Recovery strategy	*Lowest braking*		Baseline		*Highest jerk*		*Highest acceleration*		*Highest braking*		Number of pulses	Average score
						
				F	R	F	R	F	R	F	R	F	R		
12	M	An-kn	Fighting	2	2	2	2	2	2	1	0	2	2	10	1,7
18	M	Ankle	Fighting	1	2	2	2	2	2	2	0	2	2	10	1,7
7	M	Ankle	Fighting	2	2	2	2	2	2	0	0	2	2	10	1,6
17	M	Ankle	Fighting	2	2	2	2	2	2	0	0	2	2	10	1,6
11	M	Ankle	Fighting	2	2	2	2	2	2	1	0	2	0	10	1,5
9	F	Ankle	Fighting	0	2	2	2	2	2	0	0	2	2	10	1,4
16	F	Ankle	Fighting	2	2	2	2	2	2	0	0	1	1	10	1,4
24	F	Ankle	Squat-step	2	2	0	2	1	2	0	2	-	-	8	1,4
20	M	Ankle	Fighting	2	2	2	2	2	2	0	0	1	0	10	1,3
23	M	Ankle	Fighting	2	1	2	2	2	2	0	0	1	1	10	1,3
4	F	Ankle	Step	2	2	2	1	2	2	0	0	1	0	10	1,2
15	F	Ankle	Fighting	2	1	1	1	2	2	0	0	-	-	8	1,1
6	F	Ankle	Step	2	1	1	1	2	2	0	0	1	1	10	1,1
3	M	Ankle	Step	2	2	1	0	1	2	0	0	-	-	8	1
8	F	Ankle	Step	0	0	2	2	2	1	0	0	2	0	10	0,9
13	F	Ankle	Step	1	1	1	1	2	1	0	0	1	1	10	0,9
19	M	Ankle	Surfer	0	0	1	1	2	2	0	0	2	0	10	0,8
1	M	An-kn	Step	0	2	2	0	1	0	0	0	1	0	10	0,6
14	F	Ankle	Step	2	2	0	0	0	0	0	0	-	-	8	0,5
21	M	Ankle	Step	2	0	1	0	1	0	0	0	0	0	10	0,4
5	F	Ankle	Step	2	0	0	0	0	0	0	0	0	0	10	0,2
2	M	Ankle	Step	1	0	0	0	0	0	0	0	-	-	10	0,1
22	M	Ankle	Step	1	0	0	0	0	0	0	0	0	0	10	0,1
10	F	Ankle	Step	0	0	0	0	0	-	0	-	-	-	6	0
	Test performed			24	24	24	24	24	23	24	23	18	18		
	Total points			34	30	30	27	34	32	4	2	23	14		
	% Success			79	71	75	67	79	74	13	4	83	50		
	% Fail			21	29	25	33	21	26	87	96	17	50		
	% Success females			73	73	64	73	73	80	0	10	86	43		
	% Success males			85	69	85	62	85	69	23	0	82	45		
															

**FIGURE 3:**
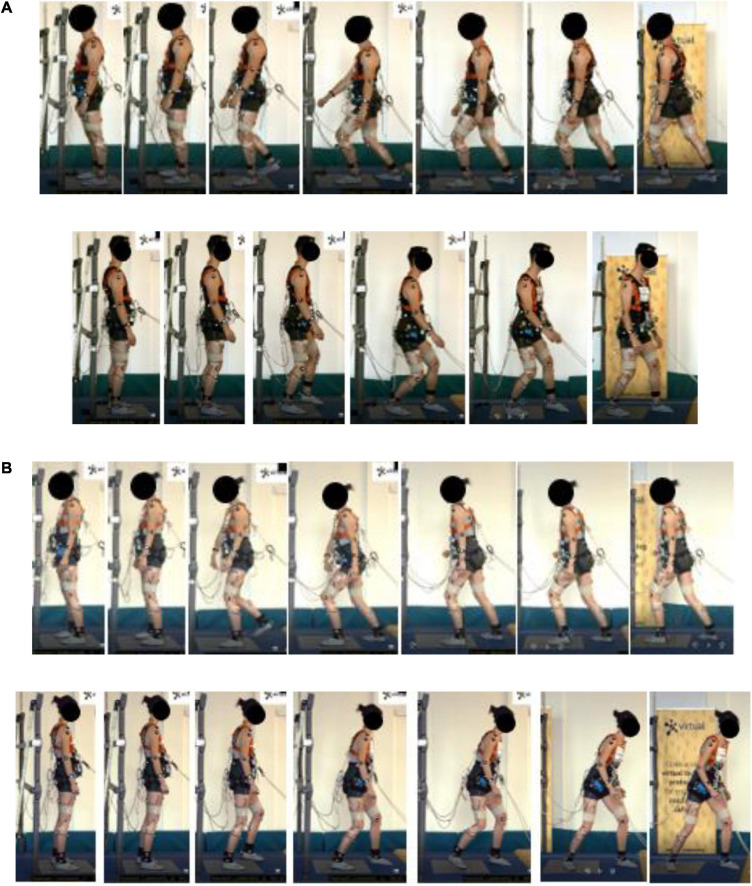
Typical forward and rearward fighting stance, **(A)** male (Volunteer 12) and **(B)** female (Volunteer 16).

**FIGURE 4:**
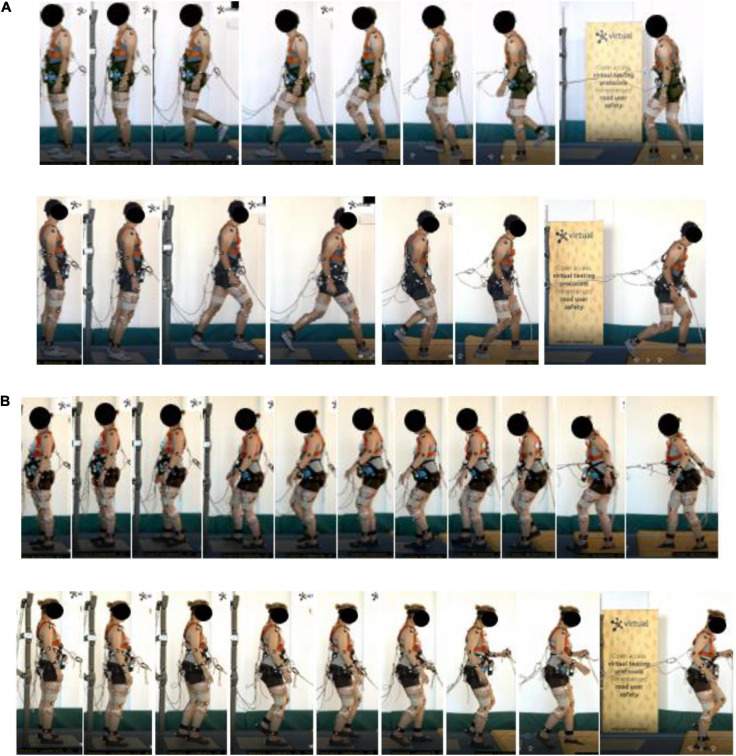
Typical forward and rearward step strategy, **(A)** male (Volunteer 22) and **(B)** female (Volunteer 10).

**FIGURE 5:**
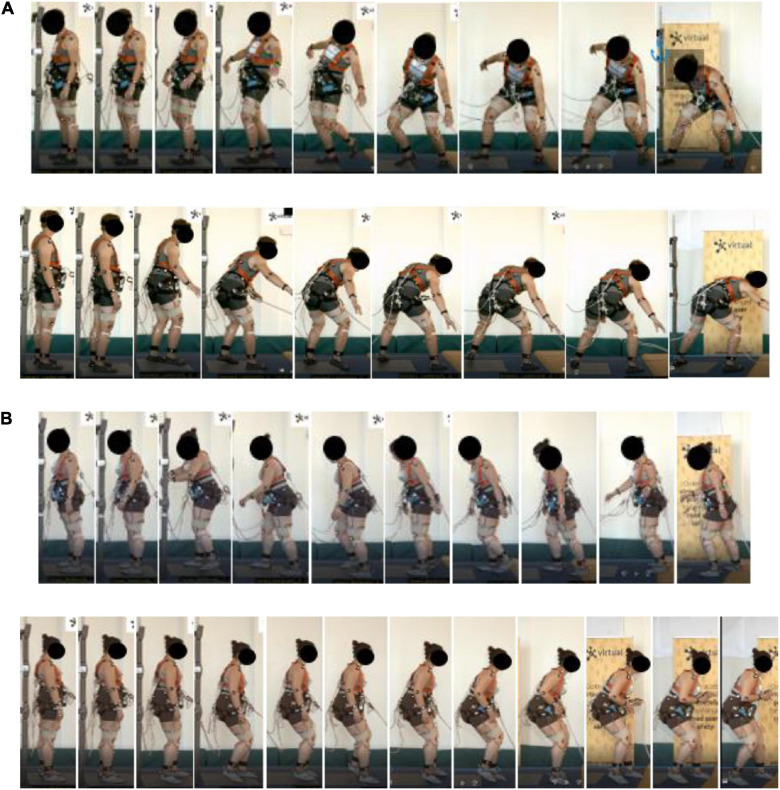
Forward and rearward, **(A)** surfer (Volunteer 19) and **(B)** squat stance (Volunteer 24).

The initial strategy was identified as the first reaction where the perturbation disturbed the balance equilibrium from the starting position. Table 5 shows that the main initial strategy for the volunteers was the ankle strategy. Knee flexion reactions were in some cases found as part of the initial strategies, indicating a knee strategy. The hip strategy was also identified as an initial strategy, although not as frequently as the ankle and knee strategies. For more severe perturbations, the step strategy was executed quicker after a brief ankle strategy, displaying the balance instability caused by the perturbation. The step strategy was identified as the most prevalent strategy to recover balance as the BoS was displaced. Two specific variants were identified, mainly continuous stepping, stretching the harness out (denoted as a pure step strategy) or a counteraction to the CIS reaction by utilizing a stance to recover a stationary BoS (denoted as the *fighting stance* later on). Figures 3–5 illustrate typical examples of the identified strategies for one perturbation, including frames from the starting position, initial strategy, and the recovery strategy phase. Variations in execution of the identified strategies are depicted in Supplementary Appendix 2,3. Section “Characterization of Identified Recovery Strategies” aims to characterize different identified fixed-support responses during the recovery phase, used for balance recovery during the step strategy, caused by a more severe perturbation. Section “Overall Description of the Execution of a Strategy” aims to characterize these variations based on their execution to provide the basis of evaluation.

#### Characterization of Identified Recovery Strategies

Three different recovery strategies were identified during the recovery phase, different from a pure step response.

The **fighting stance** strategy (see Figure 3) is characterized by positioning the lower body in a stable position utilizing a stance constituting of a front and rear leg, with the ankle, knee, and hip of the front leg flexed coupled with a slightly flexed torso. The rear leg has less ankle and knee flexion but is mostly characterized by a hip extension due to the leg position. The degree of external rotation of the ankle of the rear leg was more pronounced in some volunteers. From the video analysis, compensatory steps were included in the execution, to reach the stance. The fighting stance is also characterized by fixating a larger step, utilizing the step strategy. A fixed-support strategy, through a stable stance, is therefore obtained during the step strategy. The lower body musculature is utilized to position the body in a fixed position, i.e., a stance, by increasing knee and hip flexion in the front leg to control the CoM displacement within the BoS, utilizing the hip-extended rear leg for support. Furthermore, the rear leg executes the majority of the compensatory steps to maintain the stance, to adjust the BoS and stabilize the CoM.

The **surfer stance strategy** (Figure 5A), only utilized by Volunteer 19, resembles a surfer standing on a surfboard, and in contrast to the fighting stance it includes a larger rotation of the torso and the lower body. Should the weight of the torso be shifted to either leg, the torso rotates and leans over the front or the rear leg, whereas in the fighting stance the weight is mostly distributed on the front leg. The legs support the weight of the torso, however, compared to the fighting stance, the surfer stance can support the weight on either leg due to the stance being more symmetrical.

The **small-step squat strategy** (Figure 5B), utilized only by Volunteer 24, is characterized by a synergetic knee and hip action complex (squatting posture) with a step strategy utilizing small compensatory steps. The lower body musculature is utilized to lower the CoM accompanied by a broader stance to increase the width of the BoS. The small-step strategy keeps the feet close to the ground to maintain as close contact as possible, while taking small compensatory steps deaccelerate the BoS as the CoM is perturbed during the perturbation. From the video analyses, Volunteer 24 seemed to have a larger lower body, and her stepping seemed to be executed cautiously. The combination of smaller multiple compensatory steps with a squatting posture allowed the volunteer to increase body control (with the help of cautious stepping) and stability (maintaining a squat posture, to lower the CoM). Therefore, the small-step strategy can be used to counteract the momentum from the perturbation and withstands the perturbation by using smaller cautiously taken steps, while the squat strategy increases the stability of the CoM and BoS by lowering the CoM.

Overall, the fighting stance strategy converts balance instability (multiple-step response) into a stable stance, withstanding the need for stepping to control an unstable body by stabilizing the CoM within the BoS. Body control is increased due to the positioning of the legs, allowing discrepancies of CoM movement within a larger surface area (BoS), but with more stability due to flexible postural adjustments through multiple stepping and weight distribution advantages using both legs. The hip flexors and knee extensors (mainly quadriceps) allow the torso to be supported by a front leg positioned with a stable knee and hip flexion and dorsi-flexed ankle, acting as a weight-bearing component. The rear leg, characterized by a noticeable hip extension and slight knee flexion and dorsi- or plantar-flexed ankle for stability, activates the lower part of the posterior chain (hamstrings, gluteus muscles, and calves). This acts as the supporting part, providing the base for postural adjustments to support the weight-bearing front leg and change in the torso angle, thus lowering the CoM which increases stability.

#### Overall Description of the Execution of a Strategy

Although not displayed during every perturbation, most volunteers showed a preferred recovery strategy when their balance recovery was effective. During the video analyses, multiple volunteers displayed efforts to execute their preferred recovery strategy, despite the preferred recovery strategy being more challenging to execute successfully during the more severe perturbations. This was evident for the volunteers utilizing the fighting stance, as denoted in Supplementary Appendix 1 and Table 5. Volunteers with higher failure rates to maintain/recover balance usually used the pure step strategy, and showed no indication of trying to find a stance to stop the stepping, that would eventually induce harness deployment.

While the surfer stance strategy and the small-step squat strategy were only executed by Volunteer 19 and 24, respectively, the small-step squat strategy was executed differently in the forward and rearward perturbation. In the rearward direction, the squatting posture was more pronounced, as illustrated in Figure 5B with more flexion at the knees and the hips which resulted in more torso flexion and lower CoM. This was consistent for all rearward perturbations, with more flexion as the pulse severity increased. For the surfer stance strategy, closer to horizontal torso positioning was inspected for more severe perturbations although it was accompanied with difficulties in balance recovery. For example, during balance instability, the torso leaned forward such that Volunteer 19 lost his foothold and displayed cases where he braced using his hands to avoid falling. No clear differences in execution of this strategy were found in the video analysis.

The majority of the volunteers utilizing a step strategy as their recovery strategy presented continuous compensatory stepping throughout the perturbation, with the exception of those who managed to discontinue the stepping movements (stance) and recovered balance equilibrium as a result. No differences in execution among these users were found, specifically among genders, perturbation profiles or orientation. The initial strategy utilized was the ankle strategy, which caused larger ankle motion in the rearward perturbation and larger compensatory steps as a result.

Overall, the fighting stance was the most prevalent recovery strategy, together with the step strategy. Here, a wide variety of execution was found compared to the step strategy, as illustrated in Supplementary Appendix 2. Usually, some postural adjustments with regard to the stepping were made to find or adjust the stance as the body became unstable. Furthermore, the step length was larger compared to the step strategy users. Overall, the characterization of this strategy was very similar among the volunteers, where the final position of all users can be described using the characterization in Section “Characterization of Identified Recovery Strategies,” above. However, the transfer from stepping to the stance was different. Some volunteers displayed the execution of the stance more consistently than others as the stepping started, attempting to return to the stance in one single step, i.e., the stance was utilized to avoid further compensatory stepping as the BoS displaced from the starting position. This is illustrated in Figure 3. For other volunteers, multiple compensatory steps were utilized until the stance was found. No specific differences in the execution between genders were found.

### Balance Recovery Outcome During the Different Perturbations

The average score in Table 5 quantifies each volunteer’s overall success in recovering balance. The top scoring participants utilized the fighting stance strategy as their preferred balance recovery strategy, illustrated in Supplementary Appendix 1 and Table 5. The main recovery strategies were the fighting stance (10 out of 24) and the step strategy (12 out of 24). Based on the highest scores, seven out of 13 males utilized the fighting stance, of which five scored 2 points for most of the pulses, covering the top five ranking out of all volunteers. Only three out of 11 females utilized the fighting stance, with two of them ranking at the top of the grading table, below the five most successful male users of the strategy. For the squat-step (Volunteer 24) and surfer (Volunteer 19) recovery strategies, Volunteer 24 ranked tied among the top females (the other two had adopted the fighting stance strategy) while Volunteer 19 ranked in between the pure step strategy users, ranking low in Table 5.

The columns in Table 5 can be used to illustrate the effect of pulse shape on the volunteer response. The rearward-facing perturbations were the most severe conditions. The volunteers had higher success rates for balance during the *highest jerk* perturbations. In general, all volunteers expressed consistency in their preferred balance recovery strategy throughout all perturbations in both forward and rearward facing orientation, as described in Section “Overall Description of the Execution of a Strategy”. The top males were partly successful in the forward-facing perturbation of the *highest acceleration*, whereas the majority failed to fully recover balance. The results indicate that the *highest acceleration* was the most troublesome perturbation, with a slight disadvantage during the corresponding rearward-facing orientation (13 vs. 4% success). The highest rearward-facing braking pulse was the next troublesome perturbation (50% success). The forward-facing highest magnitude braking pulse had the highest success rate. However, due to safety considerations for some volunteers, not all volunteers participated during the *highest braking* pulses as indicated by the lower number of tests performed (18 out of 24 volunteers). The forward *highest jerk* and *lowest braking* pulse had the highest success rate including all volunteers (79% success each), with the rearward *highest jerk* and *lowest braking* pulse having a similar success rate (74 vs. 71%). Overall, the females were more successful than the males in the rearward perturbations, and vice versa. For the *highest braking*, the success rate was reversed but with very minor difference in the success rate. The number of females participating for that pulse was decreased from 11 to seven, and for the males from 13 to 11.

The volunteers preferring the pure step strategy exhibited more compensatory steps with shorter single-support phases to withstand the more severe the perturbation, which resulted in either harness deployment (0 points) or major postural adjustments to recover balance to obtain 1 point. The success rate for balance recovery based on the grading criteria was higher for the *highest jerk* compared to the *highest acceleration*. The *highest jerk* perturbation induced the fighting stance faster and more successfully to counteract and recover balance and adopting a stable stance. Furthermore, details from the video analyses show that the *lowest braking* was usually not severe enough to cause major balancing instability for the majority of the volunteers. The volunteers’ recovery strategies (mostly the fighting stance) were not challenged, and the execution was not problematic. In general, those who achieved 1 or 2 points for the *lowest braking* pulse, had little to no difficulties in balance recovery and at most exhibited only compensatory steps at the second half of the perturbation or utilized one step to find the stance. Also, the majority recovered balance fully, which was determined when a volunteer returned to the original starting position on the force plate. The surfer stance user failed in both perturbations during the *lowest braking* pulse. For the rest of the perturbations, the recovery strategies were used to counteract the perturbations, primarily for those utilizing the fighting stance. The fighting stance users required more compensatory steps to stabilize their stance since the CoM became more unstable. In addition, the *highest jerk* perturbations seemed to cause quicker transition into a successful fighting stance, as these volunteers displayed body control and stability after finding the stance and utilized very few postural adjustments, i.e., maintaining the stance. Supplementary Appendix 1 shows that all fighting stance users utilized their preferred recovery strategy during the jerk perturbations to obtain the highest grading. On the contrary, during the *highest acceleration and braking*, the fighting stance users displayed more difficulties in maintaining the fighting stance, and hence more balance instability. However, the grading demonstrates that the braking pulse was associated with a higher success rate for these volunteers.

### Statistical Results

The results of the statistical analysis in Table 6 shows that the distributions of the *fighting stance* and the pure step strategy are not from the same population. This indicates that their characteristics differ statistically, and that the *fighting stance* has an impact on the outcome regarding balance recovery. Since the test does not indicate in which way they differ, further analysis is needed, e.g., investigating the step length or margin of stability.

**TABLE 6:** Statistical results based on recovery strategy and gender.

	Strategy (fighting = 10; stepping = 12)				Gender (males = 12; females = 10)			
		
Pulse severity	Forward		Rearward		Forward		Rearward	
				
	*t*-stat	*p-value*	*t*-stat	*p-value*	*t*-stat	*p-value*	*t*-stat	*p-value*
Lowest Braking	1.924	*0.165*	6.181	0.012*	0.380	*0.537*	3.736	*0.053*
Baseline	9.177	0.002*	14.305	0.000*	1.364	*0.242*	0.249	*0.617*
Highest Jerk	9.535	0.002*	10.717	0.001*	0.037	*0.846*	0.027	*0.868*
Highest Acceleration	6.217	0.012*	^*a*^	*-*	^*b*^	-	^*a*^	-
Highest Braking	6.217	0.012*	6.199	0.012*	0.277	*0.598*	0.34	*0.559*

*Statistically significant. ^*a*^All participants lost balance. ^*b*^Few observations.

On the other hand, the results show no differences in the outcome of the balance recovery due to gender. This can be an artifact of the data since the results based on gender do not differentiate between strategies. The analysis for gender differences within strategies (fighting/stepping) could not be performed due to small sample sizes (*n* < 5).

## Discussion

A proposed methodology for in-depth analysis of identified strategies was defined, (i) to first identify the initial reaction at the starting position using a fixed-support strategy, and (ii) then the recovery phase dominated by CIS strategies. The purpose was to understand how postural balance was affected during the recovery phase (as defined in Section “Identification of Different Balance Strategies”), through identification of individual CIS strategies which would result in different balancing outcomes when subjected to different perturbations. The characterization serves to provide a description of the strategy execution to qualitatively understand the differentiation of the CIS strategies. This study is intended to be a first investigation of qualitatively evaluating if an effective CIS strategy to withstand higher severity perturbations exists. Hence, only healthy younger volunteers were included in this study to identify the upper perturbation thresholds (i.e., the limit where recovery could still be achieved) with respect to relevant acceleration and jerk magnitudes experienced on public transport.

The small-step squatting strategy, the surfer stance and the fighting stance were identified as recovery strategies different from pure stepping. The fighting stance was utilized by multiple volunteers with similar execution and high overall success rates, although not all managed the *highest acceleration*. The different strategies are briefly discussed in sections below.

### Surfer Stance Strategy

The surfer stance has not previously been defined in the literature and the term was suggested due to the similarity with the stance of surfers. However, it is debatable how applicable such a strategy would be on board a bus or tram. It was unique in this study, with only Volunteer 19 (male) displaying this strategy. From the video analyses, the risk of falling head-first would increase, as Volunteer 19 did lose footing and used his hands for support, which might increase the risk of head injuries. Therefore, it can be hypothesized that such a strategy might not be suitable inside a public transport vehicle and have unnecessary biomechanical demands for its execution. Thus, it will not be discussed further.

### Small-Step Squat Strategy

The next unique case is the small-step squat strategy, only utilized by Volunteer 24 (female, 160.0 cm, 72.6 kg). Generally, a step strategy is a countermeasure for balance instability, although from the more severe perturbation trials it has rarely been considered effective in balance recovery (as illustrated by Table 5), as most users deployed their harness. However, the small-step squat strategy increased body control and stability successfully, hence Volunteer 24 was ranked higher than the pure step strategy users, and tied with Volunteers 9 and 16 (both females utilizing the fighting stance strategy) in terms of successful outcomes. The cautious stepping might be effective to counteract the momentum caused by the perturbation, as it displayed slower stepping to withstand the perturbation, as opposed to the pure step strategy users that executed quicker steps (short single-support phase) and traveled a longer distance which deployed the harness. However, the less common anthropometry among the volunteers, with the above-mentioned combination of both strategies, could have been responsible for the successful outcome. The unique results in this study indicated that the small-step squat strategy was more effective than the pure step strategy to counteract the perturbed body movement, but the prevalence of this strategy was too low to draw any conclusions. Whether this strategy is useful for a general population needs further research, as it might be an outlier of mechanically efficient usage for this anthropometry rather than balance strategy effectiveness. As this strategy was also unique in this study, it will also not be discussed further.

### Characteristic Differences Between *Fighting Stance* and Step Strategy

The highest prevalence of utilized strategies was found for the *fighting stance* (10 out of 24) and the pure step strategy (12 out of 24). Overall, the fighting stance resulted in the highest overall scores, displaying its effectiveness in balance recovery in both its female and male users. Although this study only included 11 females and 13 males, the males seemed to execute the fighting stance more frequently compared to the females (seven males and three females). Since the fighting stance can only be successful if the perturbation can be counteracted by stopping continuous compensatory stepping, more muscular strength and body control might be required to produce the stability needed to find a stance stable enough to avoid stepping and stabilizing the CoM within a stationary BoS. Since all volunteers were subjected to the same perturbations, this might suggest that females had more difficulties in executing the fighting stance due to anthropometric aspects. For example, it was harder to execute the fighting stance during the *highest acceleration* pulse, which represents the most severe condition for the volunteers. However, some of the top-ranked males consistently adopting the fighting stance were able to withstand the *highest acceleration* in the forward direction, displaying better execution than other users of the same strategy. Despite the less successful balance recovery among the female users in this study, these females showed how effective the fighting stance was for the other perturbations in that they were more successful than the males using the pure step strategy. Karekla and Tyler (2018a) found that younger volunteers, the strongest of the sample also containing older volunteers, utilized the least effective step strategies to withstand the perturbations of that study (1.5 m/s^2^). The more effective steps were utilized by mainly male and older participants, while females were less challenged during walking which contradicted previous findings that argued that women sway more and have reduced balance (Lord et al., 1996; Hsue and Su, 2014). In the current study, there were more males than females executing the fighting stance and ultimately less females that could recover balance effectively. However, this study investigated stepping responses occurring from a stationary position as opposed to normal gait inside a moving bus and the highest effectiveness was found among males. Therefore, adopting the fighting stance might be an effective and proactive balance strategy to improve the success in recovering balance during perturbations, regardless of gender. Furthermore, the statistical analysis supports these findings, where the fighting stance does have an impact on the outcome regarding balance recovery, whereas gender does not have an impact. In other words, a female executing a given strategy (e.g., the fighting stance) would have the same outcome as a male using the same strategy. Thus, female passengers would benefit the most if they can switch from a pure stepping strategy to a stance strategy. However, the last argument is based on statistical analysis of ordinal data, and more studies are needed (quantitative and qualitative) to understand the gender influence on the execution and utilization of the fighting stance.

The fighting stance utilizes the step strategy for execution, but the step characteristics between the strategies differ. From the video analysis, *fighting stance* users executed larger steps and intended to keep the stance using compensatory steps (usually changing the step length, i.e., moving either leg), and body displacement were lower compared to pure steppers. The pure steppers executed compensatory steps with increased body displacement, rather than maintaining a stance posture and executing steps for postural adjustments which all classified *fighting stance* volunteers utilized. The effectiveness of the *fighting stance* might be due to increased body control and stability as the severity of the perturbation increases, to induce effective balance with the lower body positioning (with a leg in front of the CoM, broadening the BoS) during unexpected perturbations to lower the risk of injury. During the lowest braking perturbations, all *fighting stance* volunteers were less challenged than pure steppers (Table 5), and the *fighting stance* was more similar to a single-step strategy. The *fighting stance* users maintained postural balance with a fixed-support strategy (usually ankle, Table 5) and increased recovery through a CIS strategy using a larger step (King et al., 2005; Wu et al., 2007) towards the end of the pulse. This larger step response was characteristic among the *fighting stance* volunteers. Pure steppers executed multiple-step strategies during the same perturbation, displaying a lower perturbation threshold than *fighting stance* volunteers, even though this was the least severe perturbation in this study.

**FIGURE 6:**
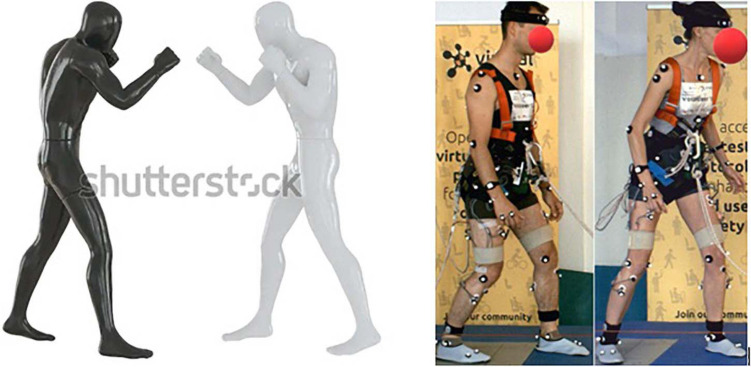
Fighting stance in martial arts (*left*) and Volunteers 12 and 16 displaying their fighting stance (*right*). Left picture from (attached link): Two Male Mannequins Black White Fighting Stock Illustration 1877892802 (shutterstock.com)–Accessed 3 Feb 2021.

With the identification of the *fighting stance*, the different characteristics can limit body displacement and increase dynamic postural stability compared to pure stepping. It provided insight on free-standing balance recovery thresholds to different magnitudes of acceleration, jerk, and braking (described in the later subsection). This stance can also be compared to an actual fighting stance, which is a stance adopted in martial arts, which might explain its effectiveness in maintaining balance as well as utilizing postural adjustments to withstand external disturbances. The lower body positioning is very similar, see Figure 6.

King et al. (2005) found that younger subjects take longer steps naturally, while older adults rather rely on shorter steps, which require less biomechanical strength. This study had a mean age of 33.8 ± 9.0 (Table 2) and therefore is not representative of the elderly. Whether the *fighting stance* is applicable and of benefit also to older adults, should be investigated further. Increasing lower body biomechanical strength (King et al., 2005; Carty et al., 2012a) might benefit older adults to execute larger steps more naturally and utilizing the single-step strategy over multiple stepping with less physical restraints. Hence, the opportunity to utilize effective recovery strategies might be possible. Previous studies (McIlroy and Maki, 1996; Hsiao and Robinovitch, 1999, 2001) have argued to not replace natural multiple stepping that occur during severe perturbations with a pure single-step response to recover balance, which ultimately can limit the perturbation thresholds, but to instead enhance (increased effectiveness) the multiple-step response (Hsiao-Wecksler and Robinovitch, 2007). Hence, it would be interesting to utilize the idea by Hsiao-Wecksler and Robinovitch (2007) to evaluate how multiple-step responses can be controlled, e.g., minimize body displacement, to improve postural control on public transport. From this, acting as one important factor among others [such as perturbation-based training (Mansfield et al., 2015)], an increased tolerance to higher perturbations could be feasible.

### Recommended Perturbation Thresholds Based on the Recovery Outcomes

The execution of the balance strategies was rapid for the *higher jerk* perturbation with effective outcome. This was not seen for the *highest acceleration*. The *highest braking* event did not include all volunteers, but the execution and the absolute outcome was not as successful as during the jerk. As seen in this study during the *higher acceleration* and *braking*, and in some of the *fighting stance* volunteers during the *highest jerk*, is that multiple compensatory steps were used to execute the *fighting stance*. Here, based on the effectiveness results in Table 5, successful recovery was less seen for the *highest acceleration* but more common for the *highest braking*, which indicates that the acceleration threshold was reached but not necessarily for the braking maneuver. This successful recovery was not seen for the pure steppers for the acceleration and braking perturbations. Multiple pure steppers were excluded from the braking pulses, the findings are mostly determined by the *fighting stance* responses. Thus, out of the higher severity pulses, the *highest jerk* perturbations might be more favorable for successful execution of balance recovery strategies compared to higher acceleration and braking.

Recent studies on bus perturbations (Karekla, 2016; Karekla and Tyler, 2018a, b; Karekla and Fang, 2021) recommended an acceleration level below 2.0 m/s^2^ to account for postural balance during gait using handrails, and 1.0 m/s^2^ without handrails. A jerk recommendation for comfort at 0.9 m/s^3^ was mentioned, but is not comparable to the findings in this study considering the recovery outcome in this study for both the baseline pulse (5.6 m/s^3^) and the *highest jerk* pulse (11.3 m/s^2^) where the majority of the volunteers managed to recover balance successfully. However, the identification of the fighting stance with resulting volunteer responses showed that the *baseline* pulse (1.5 m/s^2^) was not as problematic for this volunteer group together with the *highest jerk* and *lowest braking* pulses. Furthermore, the *baseline* pulse was too troublesome for the pure steppers, as most scored between 0 and 1 points, indicating that an acceleration of 1.0 m/s^2^ might be more realistic and improving the stepping response using the fighting stance characteristics might increase the perturbation threshold to at least 1.5 m/s^2^. It is also arguable that pure steppers should not exceed either jerk level, as their multiple step response was more unstable than volunteers with the *fighting stance*. This shows that higher perturbation thresholds can be allowed if the free-standing passengers are initially at a standstill, if *fighting stance* is utilized, but careful consideration is needed during gait such as boarding, alighting, or finding a seat inside a moving bus or tram. Avoiding pure stepping during free-standing scenarios by using the *fighting stance* might allow postural control and less body displacement, as the results in this study showed higher harness deployment rate for pure steppers (indicative of 0 points in Table 5). Providing hand support, such as handrails and/or horizontal/vertical bars, will increase the opportunity to maintain balance, as postural sway during perturbations decreases (Maki and McIlroy, 1997; Ustinova and Silkwood-Sherer, 2014; Karekla and Fang, 2021). Future studies should investigate hand support with an effective strategy, e.g., with characteristics such as the *fighting stance*, to increase the knowledge on utilizing effective CIS strategies and perturbation thresholds relevant for controlling vehicle dynamics for public transport. However, the *fighting stance* is a single-step strategy executed anteriorly to the CoM in a forward- or rearward-facing posture, which is a reasonable stepping characteristic during forward or rearward translations, but its relevancy in lateral configurations is unknown. The absence of lateral perturbations with respect to public transport needs to be addressed to complement the current study. The literature on lateral perturbations have provided more characterization by identifying different types of side-step and cross-step strategies (Borrelli et al., 2019; Batcir et al., 2020). However, recovery strategies when facing laterally to the direction of travel might induce other stepping responses when subjected to more severe perturbations as in the current study. Such literature with respect to public transport has not been found. Hence, studying acceleration and jerk perturbations in lateral-facing directions, to identify perturbation thresholds among free-standing passengers, is needed. More complex maneuvers, such as turning, should also be studied to identify and characterize stepping responses in free-standing scenarios.

## Conclusion

The qualitative investigation of identification and characterization provided insight on different CIS strategies executed during severe perturbation levels similar to those on public transport. The *fighting stance* was identified as the most effective recovery strategy to limit body displacement and increase dynamic stability during severe perturbations, compared to pure stepping. It also displayed recovery (no harness deployment) during perturbations that were more challenging for pure steppers. Thus, *fighting stance* users have higher perturbation thresholds and could withstand all perturbations, with the exception of the *highest acceleration* in both directions being too severe. A limitation in this study was the exclusion of quantitative measures, which should be utilized in conjunction with in-depth qualitative analysis (identification and characterization of the step responses), to determine the effectiveness of a balance strategy. Identifying and characterizing recovery step strategies among older adults, using lower magnitudes of acceleration, jerk, and braking, should be investigated to identify relevant perturbation thresholds. For this group, instructing the *fighting stance* and compare to pure stepping should be explored, as it might increase postural balance by increasing the effectiveness of their multiple-step responses. Overall, the *fighting stance* supports previous findings on the higher effectiveness of a single-step strategy over multiple-step strategies, since the *fighting stance* characteristics are similar to a single-step strategy but utilizes multiple stepping for postural adjustments. This shows that additional characterization provides details on how to execute an effective multiple-stepping response.

## Data Availability Statement

The raw data supporting the conclusions of this article will be made available by the authors, without undue reservation.

## Ethics Statement

The studies involving human participants were reviewed and approved by National Medical Ethics Committee, Ministry of Health, Republic of Slovenia, Štefanova 5, SI-1000 Ljubljana (http://www.kme-nmec.si/). Application number was: 0120-63/2019/4. The participants provided their written informed consent to participate in this study.

## Author Contributions

JX and AS designed the methodology and conducted the video analyses. JX analyzed the results and wrote the manuscript, with some assistance from AS. AS conducted the statistical analysis. AK, SK, RT, CK, and AL provided feedback during the video analyses. AK reviewed the methodology and improved the concepts. AS, AK, SK, RT, CK, and AL read and edited the manuscript. All the authors critically reviewed and approved the final manuscript.

## Conflict of Interest

The authors declare that the research was conducted in the absence of any commercial or financial relationships that could be construed as a potential conflict of interest.
