# Supplementary material for: Identifying and Characterizing Types of Balance Recovery Strategies Among Females and Males to Prevent Injuries in Free-Standing Public Transport Passengers
Source: Front Bioeng Biotechnol. 2021 Jul 5;9:670498. doi: 10.3389/fbioe.2021.670498 (PMC8287834; doi:10.3389/fbioe.2021.670498)

**Appendix 1. Identified balance recovery strategies for each perturbation trial. The overall initial and recovery strategies are also tabulated. Abbreviations: an – ankle, kn – knee, hi – hip, sp – step, fi – fighting stance, sq – squat, su – surfer, n.a. – not applicable.**

| **Volunteers** | ***Lowest braking*** | | ***Baseline*** | | ***Highest Jerk*** | | ***Highest Acceleration*** | | ***Highest Braking*** | | **Initial** | **Recovery** |
| --- | --- | --- | --- | --- | --- | --- | --- | --- | --- | --- | --- | --- |
|  | **F** | **R** | **F** | **R** | **F** | **R** | **F** | **R** | **F** | **R** | **Strategy** | **Strategy** |
| 1 (M) | An\|n.a. | Kn-An \| Fi | Kn-An\|Fi | An\|Sp* | Kn-An\|Sp | An\|Sp* | Kn-An\|Sp* | An\|Sp* | Kn-An\|Fi | An-Hi\|Sp* | An-Kn | Step |
| 2 (M) | An-Hi\|n.a. | An\|Sp* | An\|Sp* | An\|Sp* | An\|Sp* | An\|Sp* | An\|Sp* | An\|Sp* | n.a. | n.a. | Ankle | Step |
| 3 (M) | An\|n.a. | An\|Sp | An-Kn\|Sp | An\|Sp* | An-Kn \|Sp | An\|Fi | An-Kn \|Sp* | An\|Sp* | n.a. | n.a. | Ankle | Step |
| 4 (F) | An\|Sp | An\|Sp | An\|Sp | An\|Sp | An-Hi\|Fi | An\|Fi | An-Kn\|Sp* | An\|Sp* | An-Kn\|Sp | An\|Sp* | Ankle | Step |
| 5 (F) | An\|Sp | An\|Sp* | An\|Sp* | An\|Sp* | An\|Sp* | An\|Sp* | An\|Sp* | An\|Sp* | An\|Sp* | An\|Sp* | Ankle | Step |
| 6 (F) | An\|Kn | Kn-An\|Sp | An-Kn\|Sp | An\|Sp | An-Kn\|Fi | An\|Sp | An-Kn\|Sp* | An\|Sp* | An-Kn\|Sp | An\|Sp | An-Kn | Step |
| 7 (M) | An\|Sp | An\|Fi | An\|Fi | An\|Fi | An\|Fi | An\|Fi | An\|Sp* | An\|Fi* | An\|Fi | An\|Fi | Ankle | Fight |
| 8 (F) | An\|n.a. | An\|Sp | An\|Fi | An\|Sp | An\|Fi | An\|Sp | An\|Sp* | An\|Sp* | An\|Fi | An\|Sp* | Ankle | Step |
| 9 (F) | Kn-An\|n.a. | An\|Sp | An\|Fi | An\|Fi | Hi-An\|Fi | An\|Fi | Kn-An\|Sp* | An-Kn\|Fi* | An\|Fi | An\|Fi | Ankle | Fight |
| 10 (F) | An\|Sp | An\|Sp* | An\|Sp* | An\|Sp* | An\|Sp* | n.a. | An\|Sp* | n.a. | n.a. | n.a. | Ankle | Step |
| 11 (M) | Kn\|Kn | An\|Fi | An-Hi\|Fi | An\|Fi | An-Hi\|Fi | An\|Fi | An-Kn\|Sp | An\|Fi* | An-Kn\|Fi | An\|Sp* | Ankle | Fight |
| 12 (M) | An-Kn\|Fi | An\|Fi | An-Kn\|Fi | An\|Fi | An-Kn\|Fi | An\|Fi | An-Kn\|Fi | Kn-An\|Fi* | An-Kn\|Fi | Kn-An\|Fi | An-kn | Fight |
| 13 (F) | An-Hi(?)\|Sp | Hi-An\|Sp | An\|Sp | An\|Sp | An\|Sp | An\|Sp | An\|Sp* | An\|Sp* | An\|Sp | Hi\|Fi | Ankle | Step |
| 14 (F) | An-Sp | An-Hi\|Sp | An\|Sp* | An\|Sp* | An\|Sp* | An\|Sp* | An\|Sp* | An\|Sp* | n.a. | n.a. | Ankle | Step |
| 15 (F) | Kn-Kn | Hi-An-Kn\|Sp | An\|Fi | Hi\|Fi | An\|Fi | An\|Fi | An\|Sp* | An\|Sp* | n.a. | n.a. | Ankle | Fight |
| 16 (F) | An-Fi | Kn-Sp\|Fi | An-Kn\|Fi | An\|Fi | An\|Fi | An\|Fi | An-Kn\|Sp* | An-Hi\|Sp* | Kn\|Fi | Kn\|Fi* | Ankle | Fight |
| 17 (M) | - | An\|Fi | An\|Fi | An\|Fi | An\|Fi | An\|Fi | An\|Sp* | An\|Sp* | An\|Fi | An\|Fi | Ankle | Fight |
| 18 (M) | Sp\|Fi | Sp\|Fi | An-Sp\|Fi | An\|Fi | An\|Fi | An\|Fi | An\|Fi | An\|Fi* | An\|Fi | An-Kn\|Fi | Ankle | Fight |
| 19 (M) | Hi-An\|Sp* | Sq\|Su* | An\|Su | An\|Su | An\|Su | An\|Su | Kn-An\|Su* | An\|Su* | An\|Su | -\|Su* | Ankle | Surfer |
| 20 (M) | An\|Fi | An\|Fi | An\|Fi | An\|Fi | An\|Fi | An\|Fi | An\|Fi* | An\|Sp* | An\|Sp | An\|Sp* | Ankle | Fight |
| 21 (M) | An\|Fi | An\|Sp* | An\|Sp | An\|Sp* | An\|Sp | An\|Sp* | An\|Sp* | An\|Sp* | An\|Sp* | An\|Sp* | Ankle | Step |
| 22 (M) | An\|Sp | An\|Sp* | An\|Sp* | An\|Sp* | An-Kn\|Sp* | An\|Sp* | An\|Sp* | An\|Sp* | An-Kn\|Sp* | An\|Sp* | Ankle | Step |
| 23 (M) | An\|Sp | An\|Sp | An\|Fi | An\|Fi | An\|Fi | An\|Fi | An\|Fi* | An\|Fi* | An\|Fi | An\|Fi | Ankle | Fight |
| 24 (F) | An\|Fi | An\|Sp | n.a. | Hi-Kn\|Sq-Sp | Hi\|Sq-sp | An\|Sq-sp | Hi\|Sq-sp | An\|Sq-sp | n.a. | n.a. | Ankle | Squat-step |

**Appendix 2. Fighting stance variations among volunteers.**


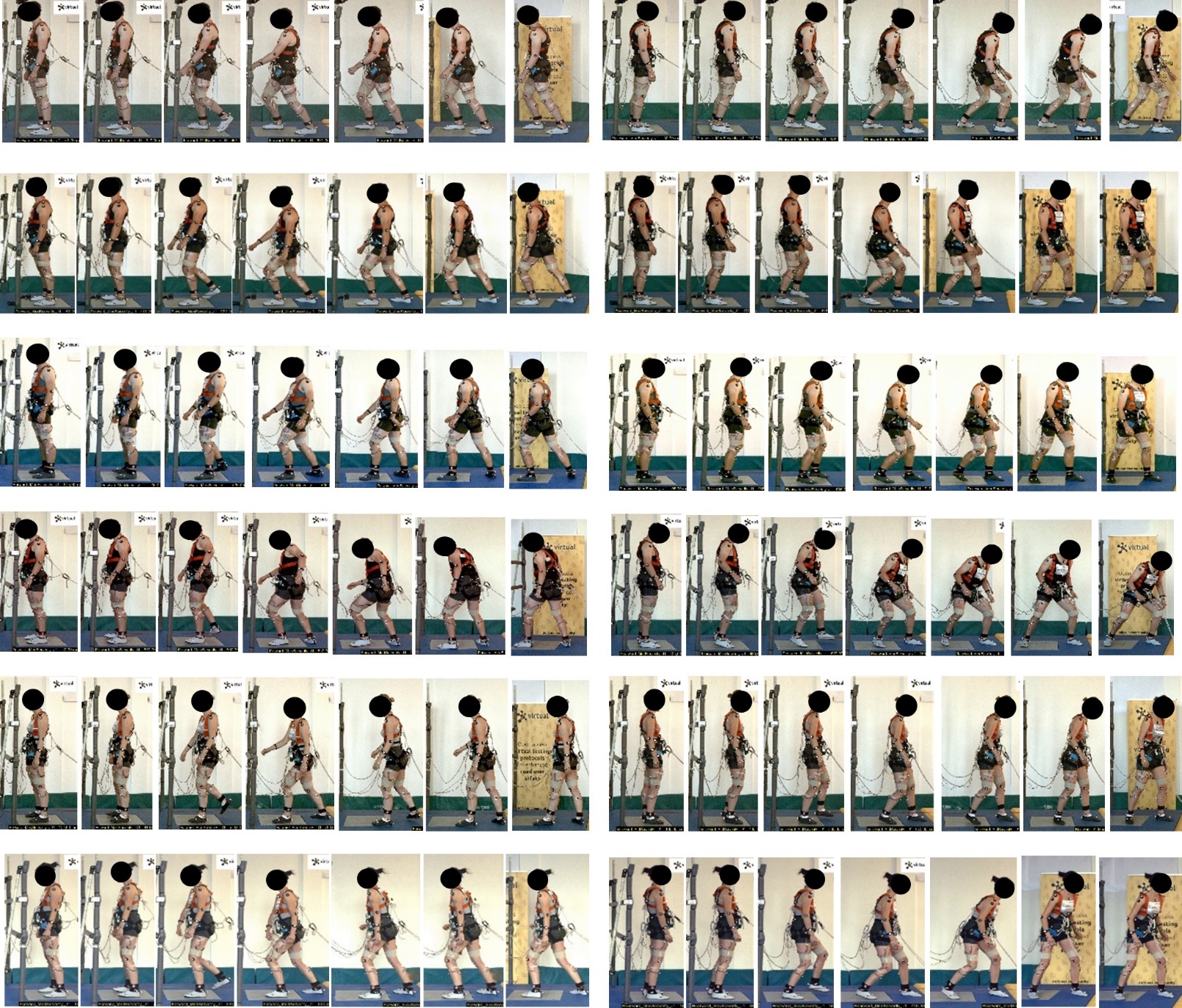


**Appendix 3. Stepping variations among volunteers.**


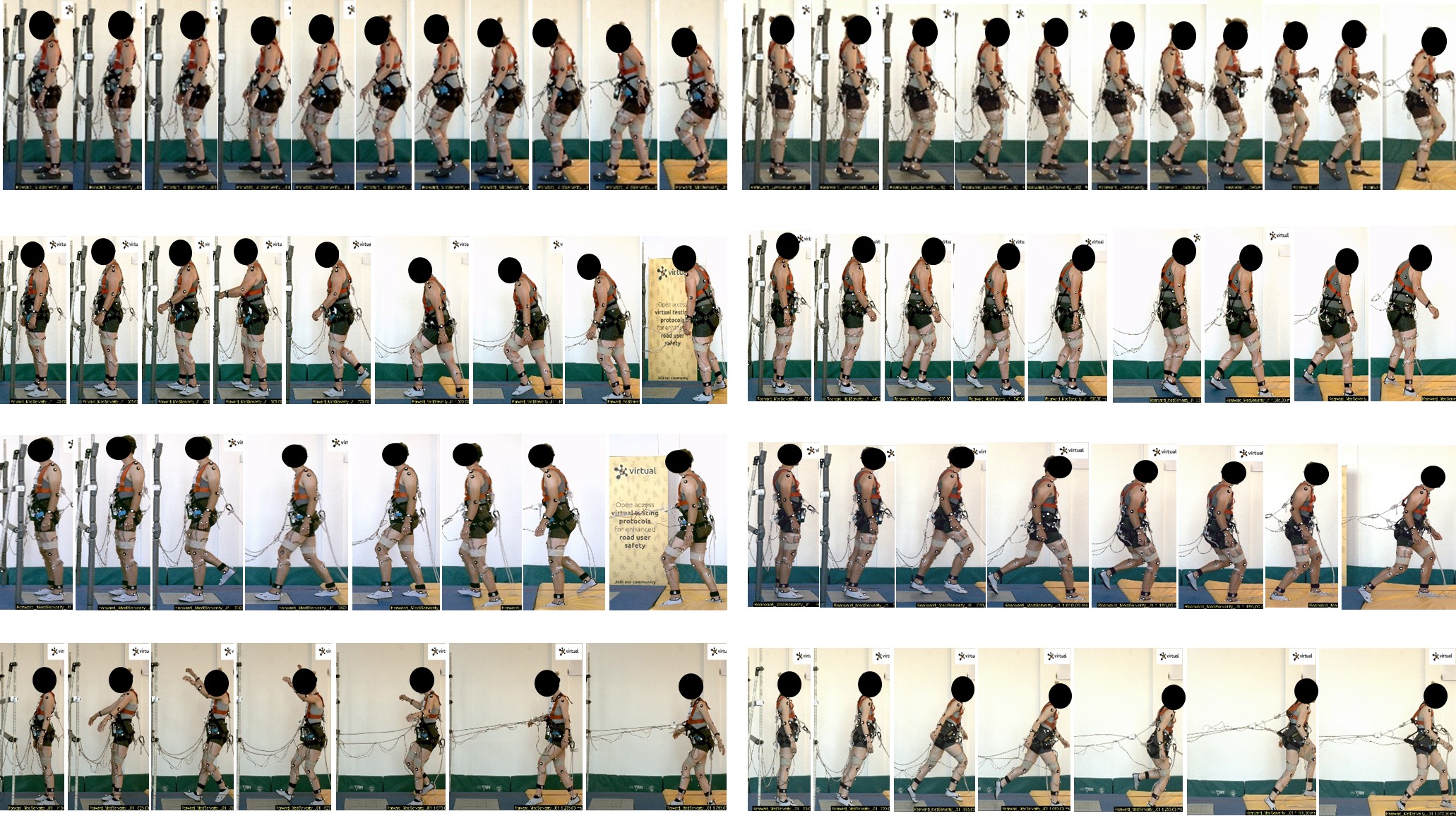

Supplement: Supplementary file 1 [file Data_Sheet_1.docx]
